# Influence of cartilage defects and a collagen gel on integrity of corresponding intact cartilage: a biomechanical in-vitro study

**DOI:** 10.1007/s00402-024-05530-z

**Published:** 2024-10-14

**Authors:** Alexander M. Pieringer, Stefan Milz, Andreas B. Imhoff, Stephan Vogt

**Affiliations:** 1 Department of Sports Orthopaedics, Technical University of Munich, Klinikum rechts der Isar, Munich, Germany; 2 Department of Anatomy, Ludwig Maximilians University Munich, Munich, Germany; 3 Department of Sports Orthopaedics, Hessing Stiftung Augsburg, Augsburg, Germany

**Keywords:** Articular cartilage, Focal lesion, Cartilage damage, Cartilage repair, Biomaterial

## Abstract

**Introduction:**

Numerous cartilage repair procedures have been developed for focal lesions to minimize suffering and possibly prevent the development of osteoarthritis with a focus on so-called one-step procedures. The aim of this work was to investigate the effects of both focal cartilage defects and a biomaterial (ChondroFiller) on the corresponding articular cartilage.

**Materials and Methods:**

On a friction test stand, 18 porcine osteochondral cylinders were tested in six experimental setups under cyclic loading (33 N) against a friction partner in saline solution. The friction partner (cartilage, bone, cartilage defect, cartilage defect with ChondroFiller) and the running times (1 hour and 6 hours) were varied. The damage to the osteochondral cylinders was assessed histologically using a visual damage classification.

**Results:**

The cartilage versus bone group showed severe cartilage damage in both the one-hour and six-hour experiments, with an average damage score of 3.5. Damage in the cartilage versus cartilage defect group was moderate, with damage values of 2.5 (1 h) and 2.67 (6 h). The cartilage versus cartilage defect with ChondroFiller group showed a damage value of 2.67 for the one-hour and 2.5 for the six-hour trials.

**Conclusions:**

Even focal grade IV cartilage lesions can lead to significant damage to the corresponding cartilage in vitro. The damage could not be reduced by the use of ChondroFiller, likely because of the initial instability of this biomaterial. Therefore, a biomaterial must be stable in the beginning with regard to full weight-bearing, or joint loading should be delayed until stable filling of the defect is achieved.

## Introduction

Pain, swelling, functional limitations: the complaints from which patients with focal cartilage damage may suffer are manifold [1]. But it is not only patient quality of life that sometimes decreases drastically. Focal cartilage damage can also pose a serious threat to the affected joint, sometimes leading to osteoarthritis [2]. Even more, numerous scientists are currently striving to optimize current cartilage therapy methods and to develop new therapeutic approaches. Especially in young patients, intensive efforts are being made to prevent progression to osteoarthritis and to minimize suffering. While prosthetic treatment becomes increasingly important with advancing age, there are some biological procedures for the treatment of focal cartilage damage for patients up to the age of 40. A common cartilage therapy for this purpose is, for example, matrix-associated autologous chondrocyte transplantation (MACT), in which the patient’s own cartilage cells are harvested in a first step so that they can be re-implanted on a cell carrier after multiplying by the millions [3]. Currently, one-step procedures such as minced cartilage are also becoming more and more interesting, since only one operation is necessary, which reduces the risk of complications [4]. There are numerous studies demonstrating the clinical efficacy of these biological cartilage therapy procedures [5–8]. However, in which way the different biomaterials directly affect the quality of the opposing articular cartilage has been poorly studied. There is also a lack of work examining what damage focal cartilage damage can cause to the opposing cartilage. Instead, the existing literature is mostly concerned with the effects of focal cartilage damage on the biomechanical properties of the cartilage immediately adjacent to it [9, 10]. Therefore, in this work, the focus was on the corresponding cartilage. The influence of cartilage defects and biomaterials on the opposite cartilage were investigated in a friction test stand using porcine knee specimens. This experimental setting (testing of porcine knee preparations in the friction test stand) has already proven successful in two other studies, which were also carried out at our institute. In one work, the influence of meniscal sutures on the corresponding cartilage was investigated [11], while the other dealt with the effects of metallic implants on cartilage [12].

We assume that a cartilage defect also can cause relevant damage to the opposite cartilage. Furthermore, we expect that the cartilage damage will be minimized by the use of a biomaterial like *ChondroFiller*.

## Methods

### Study design

On a friction test stand, 18 porcine osteochondral cylinders were tested in six experimental setups under cyclic loading (33 N) against a friction partner in saline solution. The friction partner (bone, cartilage defect, cartilage defect with *ChondroFiller*) and the running times (1 h and 6 h) were varied. The damage of the osteochondral cylinders was assessed histologically by using a visual damage classification.

**Fig. 1:**
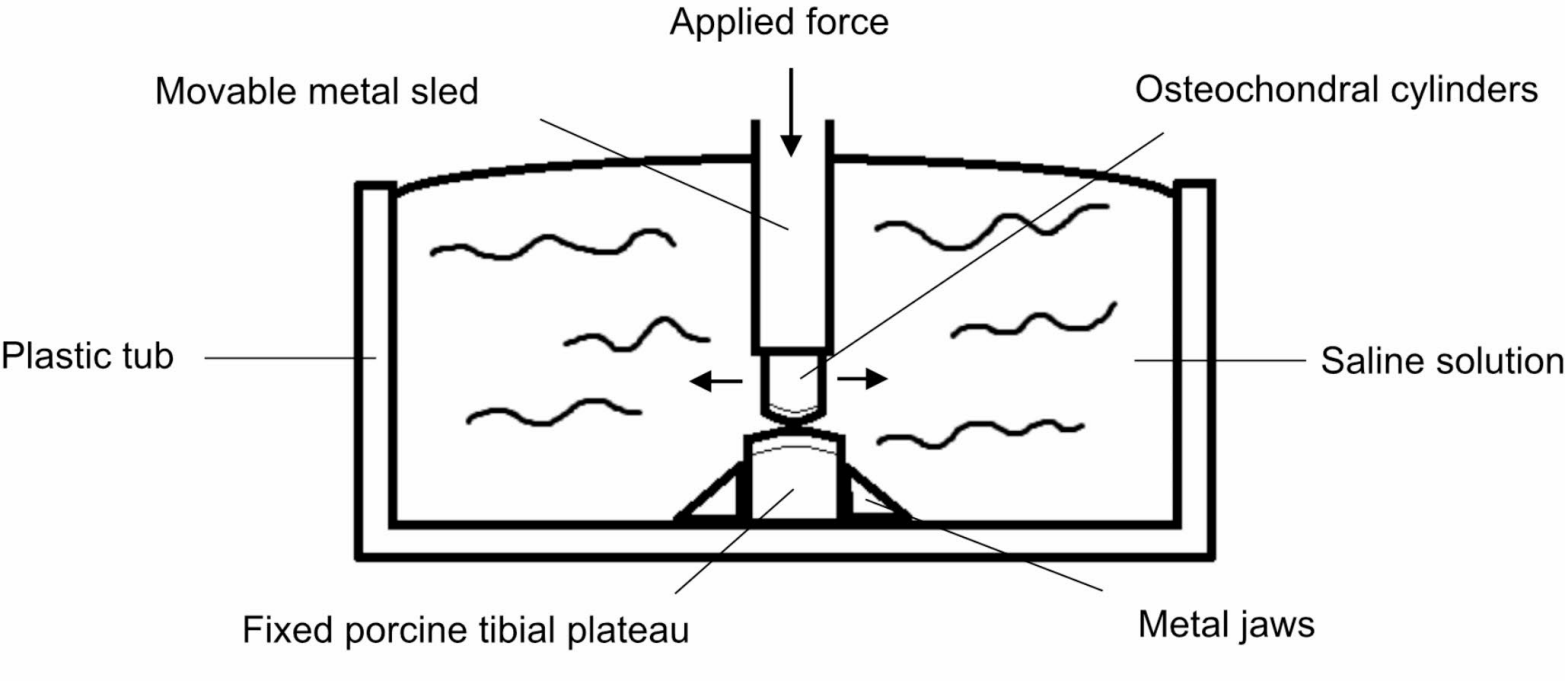
Schematic drawing of the friction test stand

A total of 18 fresh frozen porcine knees (age between 5 and 7 months, weight about 80 ± 8.5 kilos) were used to perform this study, that were collected from the slaughterhouse immediately after the slaughtering process. After knees removal from the animals, the soft tissue were removed carefully and stored at -20 °C. Before testing, the knees were thawed overnight at room temperature. Osteochondral cylinders with a diameter of 10 mm were harvested from the center of the medial femoral condyle according to manufacturer’s instructions (OATS^®^ system, Arthrex, Florida, USA) and installed in the friction test stand (Fig. 1).

Porcine tibial plateaus were used as friction partners, that were first prepared for each test group (bone, cartilage defect, cartilage defect with gel*)* and then fixed between two metal jaws in the friction test stand. To create a standardized cartilage defect (6 mm) on the medial tibial plateau, the Osteochondral Autotransfer System (OATS^®^ System, Arthrex, Florida, USA) was used to remove the cartilage. *ChondroFillerliquid* (Amedrix GmbH, Esslingen, Germany) was inserted into the cartilage defect according to the manufacturer’s instructions.

Three osteochondral cylinders per test group were tested for 1–6 h each (total number of osteochondral cylinders *n* = 18). Physiological saline solution was used as the test medium in each case. Table 1 provides an overview of the test setups.

**Table 1:** Overview of the test groups

*Group (n = 3 cylinders per group)*	Abbreviation
Cartilage against bone for 1 h	*C-B 1 h*
Cartilage against bone for 6 h	*C-B 6 h*
Cartilage against cartilage defect for 1 h	*C-CD 1 h*
Cartilage against cartilage defect for 6 h	*C-CD 6 h*
Cartilage against cartilage defect with gel for 1 h	*C-CD gel 1 h*
Cartilage against cartilage defect with gel for 6 h	*C-CD gel 6 h*

After completion of the friction tests, two histological preparations of each osteochondral cylinder were made and the cartilage damage was determined microscopically.

### Friction test stand

**Fig. 2:**
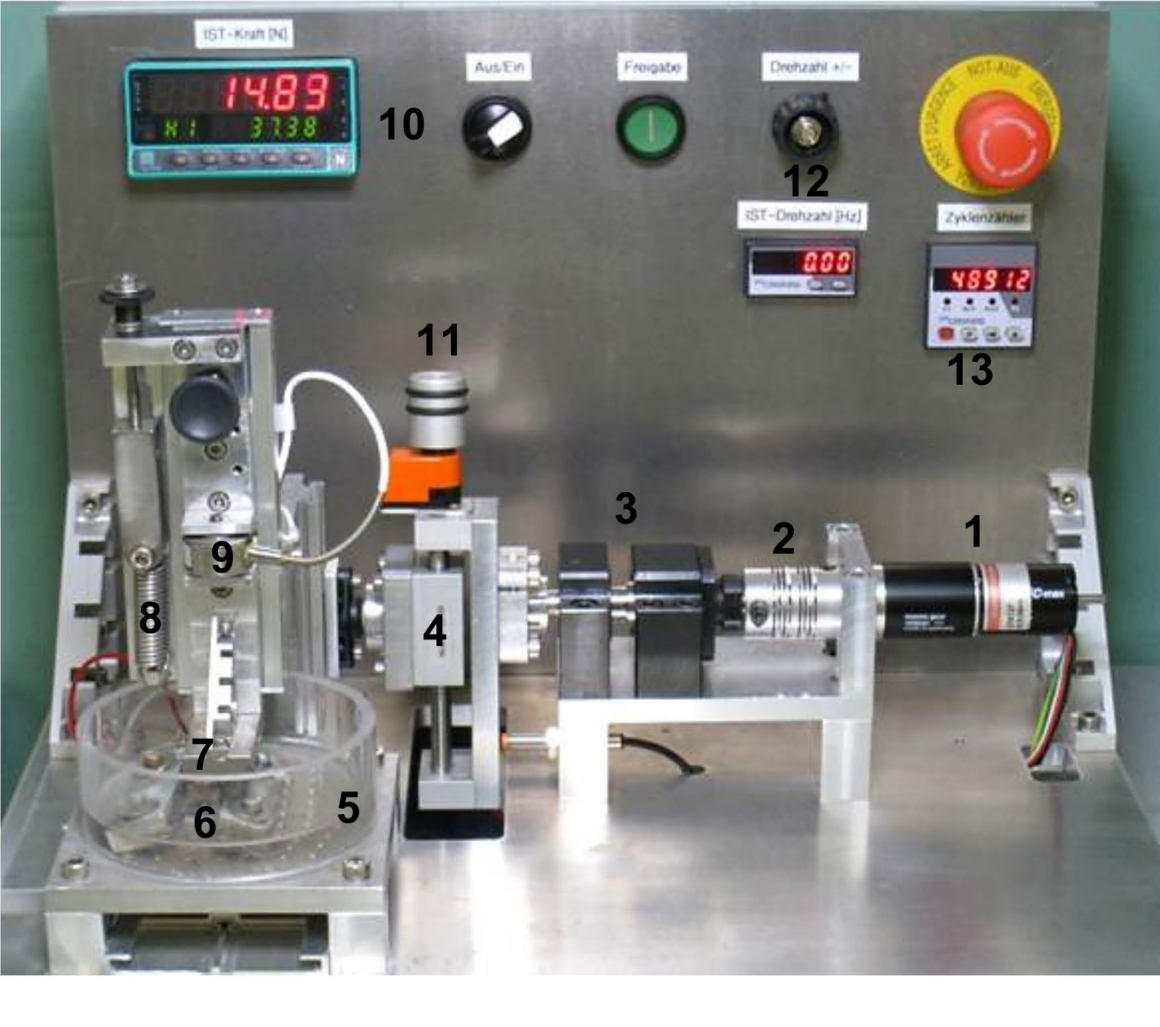
Setup of the friction test stand; (1) Drive mechanism, (2) Axis of rotation, (3) Axle box, (4) Eccentric tappet, (5) Plastic tub, (6) Fixed specimen, (7) Mobile specimen, (8) Tension spring, (9) Force sensor, (10) Force display, 11) Setting length amplitude, 12) Rotational speed, 13) Cycle counter

The friction test stand uses a drive mechanism and a fixed base to test two friction partners and was specifically designed to perform cyclic continuous loading (Fig. 2).

The porcine tibia plateaus were clamped between the metal jaws of the base plate. The osteochondral cylinders, on the other hand, served as movable friction partners. An axis perpendicular to the linear axis preloads the specimen. A tappet converts an electric motor’s rotatory action into a linear movement for the specimen. The linear motion is adjustable from 2 to 20 mm (stroke = 40 mm) at 0.5 to 2.25 Hz (Hertz). A serial setting with a force sensor (maximum load 200 N (Newton), Type 8431 − 5200, Burster Gernsbach/Germany), adjustable screw, and spring applies a continuous preload. Screw couplings fix specimens to stiff adaptor plates. A clear cylindrical dish enclosing the friction partners is utilized for liquid experiments (0.9% NaCl). Control panels can set cycle frequency, number of cycles (ZX122, Motrona, Rielasingen/Germany), and applied force (tare function, real and absolute peak force).

For this work, the parameters were chosen according to the study by Venjakob et al. [11]. In this study, the test load was calculated as a function of pig weight, the articular surface area of a pig knee, and the diameter of the osteochondral cylinder (10 mm), and was calculated to be 33 N. This resulted in a stress rate of 0.42 MPa (megapascal; 1 MPa = 1 Million Pa = 1 N/mm^2^). Since the pigs in this work were of similar weight, a test load of 33 N was also used for the experiments. The chosen cycle frequency was 1 Hz, adapted to normal walking. The sliding amplitude was 10 mm.

### Preparation and evaluation of histological preparations

**Fig. 3:**
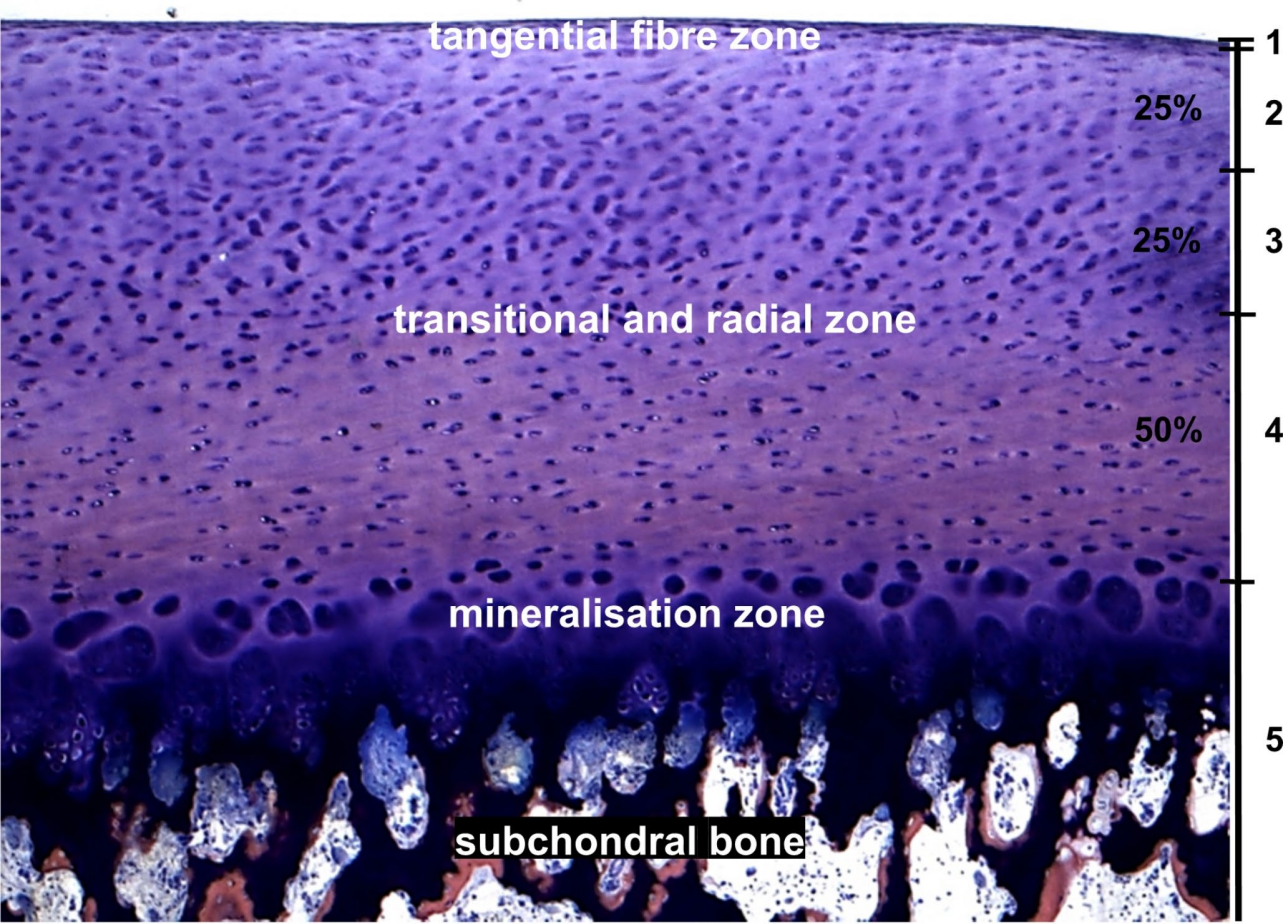
Damage table for the evaluation of histological specimen: 0 = No damage visible; 1 = Damage in the tangential fiber zone; 2 = Damage in up to 25% of the transitional and radial zone; 3 = Damage in up to 50% of the transitional and radial zone; 4 = Damage in up to 100% of the transitional and radial zone; 5 = Damage in up o at least the mineralization zone

Two sections of approximately 200 μm thickness were made from the center of the osteochondral cylinder of the MMA-embedded specimens under continuous cooling with water using a sawing microtome (SP 1600, Leica, Nussloch, Germany) with a diamond cutting blade and glued onto a plastic slide. The specimens were then ground to a thickness of 100 μm, polished with a grinding machine and, if necessary, additionally polished by hand with diamond powder. Giemsa-eosin staining was chosen as the staining method, as it allows the individual components of the bone and cartilage tissue to be shown and delineated well. The stained slides were evaluated using a reflected light microscope (Axiophot, Zeiss, Oberkochen, Germany). The slides were assessed by two persons independently at different times in a blinded manner. Focus was placed on the quantification of cartilage damage. It was performed using a damage classification (Fig. 3) based on studies by Milz, Putz and Glaser [13, 14].

## Results

Each series of experiments was performed three times with different osteochondral cylinders (*n* = 3) and two histological preparations of each were subsequently prepared and histologically evaluated. Both preparations of a specimen were assigned the same damage value in each case despite blinded assessment. In turn, 14 of the total 18 samples were assessed by the two assessors with the same damage value. In four specimens, the damage value differed by one point. In these cases, the samples were assigned the mean of the two damage values. A cartilage versus cartilage in saline solution test series served as the control group. These results are already known from another study [11]. Here, the test was carried out on the same friction test regime, also for 1 and 6 h in saline solution. Therefore, this experimental group was not rerun in this work.

Table 2 shows the damage values of the three specimens per experimental group as well as the results of the experimental group cartilage versus cartilage in saline solution by Venjakob et al. [11].

**Table 2:** Damage values of the three specimens per experimental group

Group	Specimen 1	Specimen 2	Specimen 3
*C-C 1 h*	0	0	0
*C-C 6 h*	0	0	0
*C-B 1 h*	3.5	3.5	4
*C-B 6 h*	3	4	3
*C-CD 1 h*	2	3	2.5
*C-CD 6 h*	3	3	2
*C-CD CF 1 h*	3	3	2
*C-CD CF 6 h*	2	2.5	3

Abbreviations: C = cartilage; B = bone; CD = cartilage defect; CF = ChondroFiller

Damage values: 0 = No damage visible; 1 = Damage in the tangential fiber zone; 2 = Damage in up to 25% of the transitional and radial zone; 3 = Damage in up to 50% of the transitional and radial zone; 4 = Damage in up to 100% of the transitional and radial zone; 5 = Damage in up to at least the mineralization zone

No damage occurred in the cartilage versus cartilage control group, the majority of the other preparations showed significant cartilage damage. Two third of these showed a damage score of 3 or more.

Table 3 shows the mean damage value per experimental group per runtime, as well as the overall mean damage per group.

**Table 3:** Mean damage value per experimental group per runtime and overall mean damage per group

Group	Damage value per runtime	Mean
*C-C 1 h*	0	0
*C-C 6 h*	0	
*C-B 1 h*	3.67	3.5
*C-B 6 h*	3.33	
*C-CD 1 h*	2.5	2.58
*C-CD 6 h*	2.67	
*C-CD CF 1 h*	2.67	2.58
*C-CD CF 6 h*	2.5	

Abbreviations: C = cartilage; B = bone; CD = cartilage defect; CF = ChondroFiller.

Damage values: 0 = No damage visible; 1 = Damage in the tangential fiber zone; 2 = Damage in up to 25% of the transitional and radial zone; 3 = Damage in up to 50% of the transitional and radial zone; 4 = Damage in up to 100% of the transitional and radial zone; 5 = Damage in up to at least the mineralization zone.

**Fig. 4:**
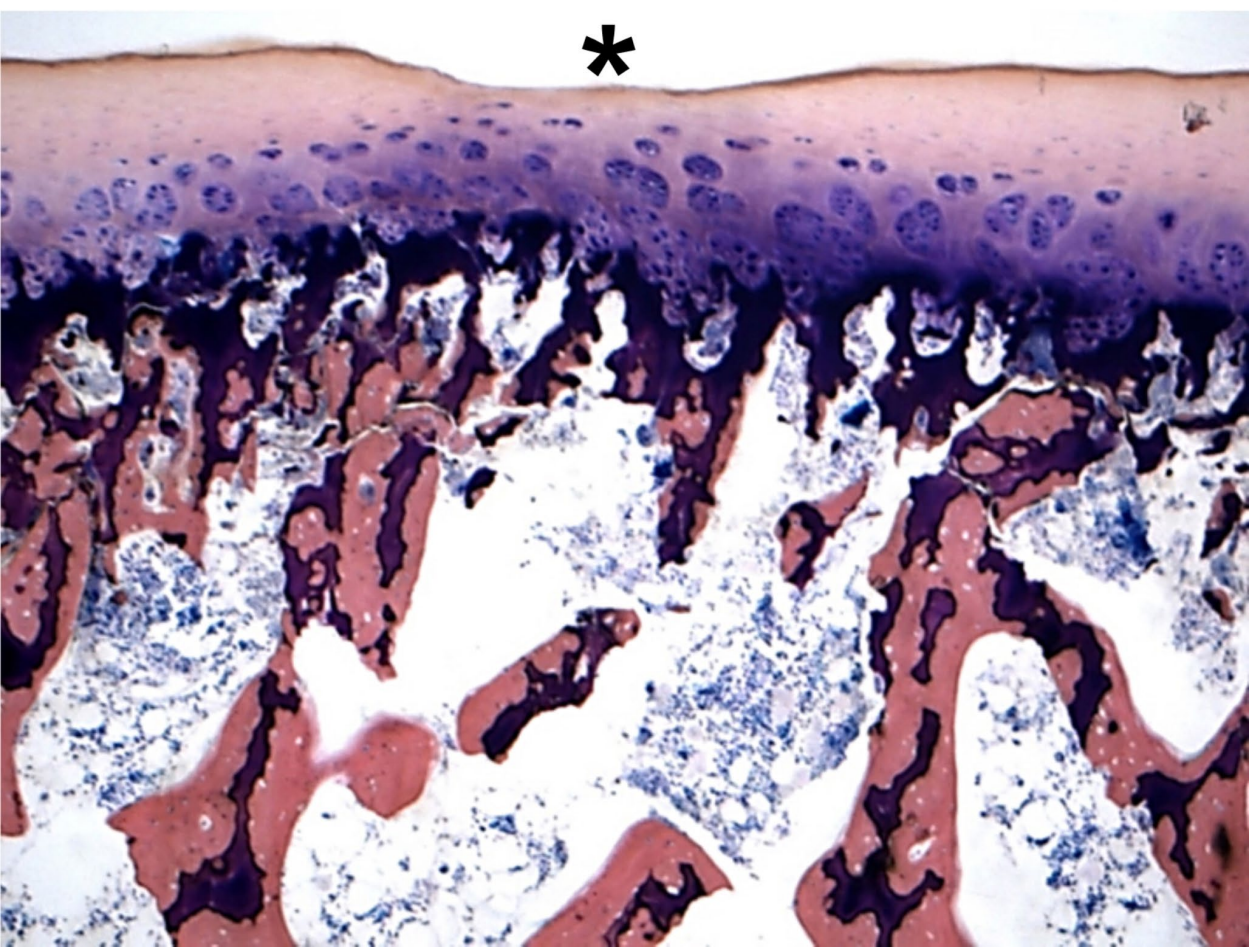
Histologic picture of the specimen 2 of the test series C-B 6 h with a cartilage defect up to 100% of the transitional and radial zone (grade 4; asterix). (Giemsa 5x)

**Fig. 5:**
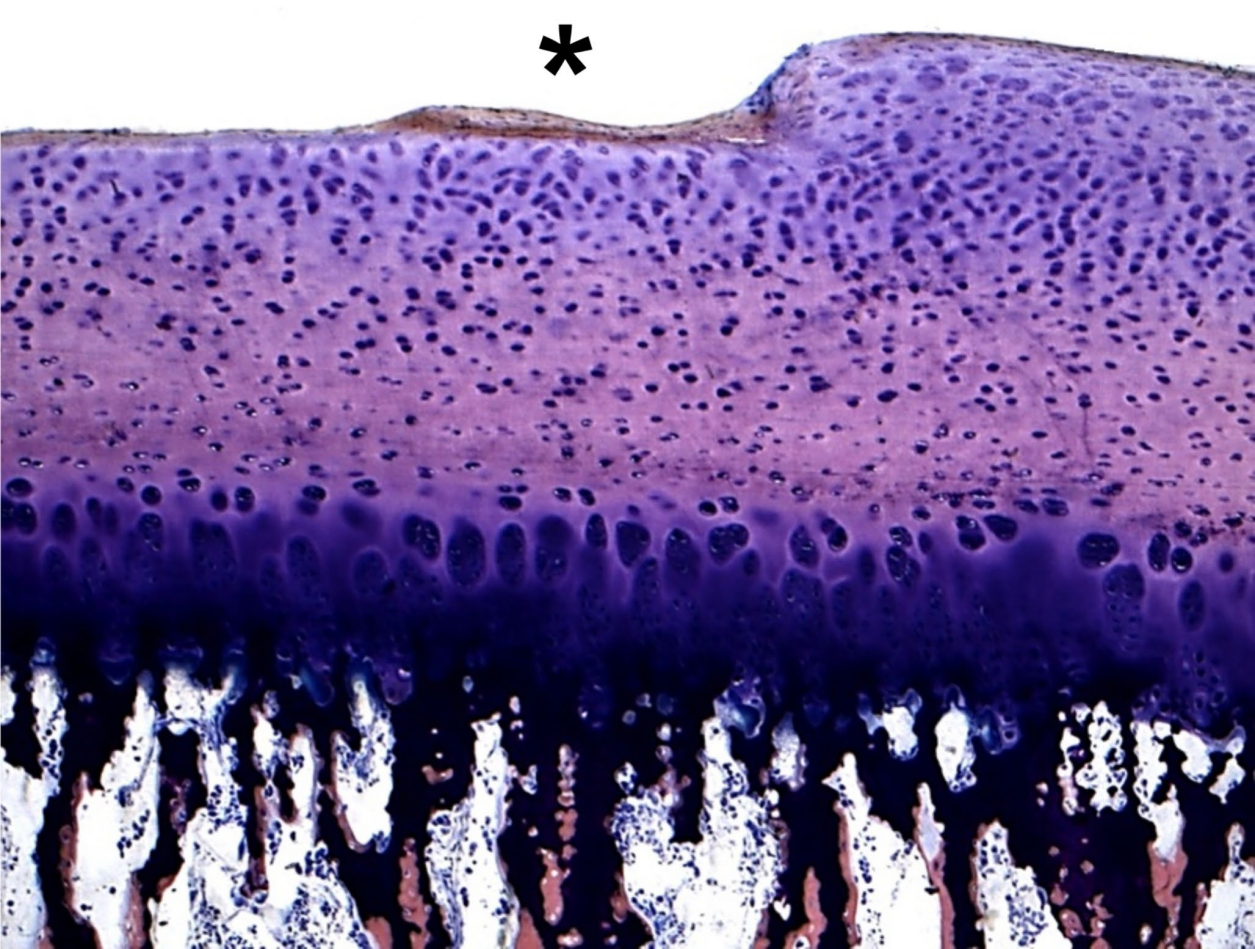
Histologic picture of the specimen 3 of the test series C-CD 1 h with a cartilage defect of the upper 25% of the transitional and radial zone (grade 2–3; asterix). (Giemsa 5x)

Figures 4 and 5 show examples of histological sections of the osteochondral cylinders with visible cartilage defects (asterix).

## Discussion

### Comparison of the run times

We initially assumed that a longer run time would lead to greater cartilage damage, as it is exposed to the load 6 times longer. In addition, Forster and Fisher were able to show in 1996 when testing bovine cartilage-bone cylinders that the coefficient of friction increases significantly with increasing test duration in NaCl.

Surprisingly, however, there was no significant difference in the damage value for the different running times (1 h vs. 6 h). The damage therefore already occurred within the first hour, although contrary to our assumption, the longer running time did not lead to a further increase in cartilage damage. Whether this would be different with a lower load would have to be investigated in further studies.

### Comparison of the groups

While the control group cartilage versus cartilage showed no cartilage damage, the total damage score in the cartilage defect versus cartilage group of 2.58 was almost a full point lower than in the cartilage versus bone group (score 3.5), but even focal cartilage damage was thus already sufficient to destroy up to 50% of the counter cartilage in these cases. Such cartilage damage can already have a substantial impact on the integrity of the remaining cartilage tissue. Several studies have shown that there can be significant increases in tensile and compressive loads in the area of the defect rim, which can result in further tissue damage [9, 15–19]. Various studies suggest that focal cartilage defects even increase the risk of developing osteoarthritis [20–23].

We assume that the sharp rim of the cartilage defect has led to damage to the opposing cartilage. This is because a cartilage defect not only appears to have a negative effect on the biomechanical properties of the surrounding cartilage, but also leads to increased stress and deformation of the opposing cartilage [15, 16]. The morphology of the cartilage defect also appears to play a decisive role. Interestingly, defects with a very vertical defect edge (as in our study) appear to cause greater deformation of the opposing cartilage than cartilage defects with a more beveled defect edge [24].

The use of biomaterials fills the cartilage defect, which is why we assumed that consequently the edge effect would be reduced and thus the opposite cartilage damage would also be less severe. *ChondroFiller* is a product of Meidrix Biomedicals GmbH (former Amedrix GmbH, Esslingen, Germany) and is offered as a form-stable gel (*ChondroFillergel*) or in a liquid form (*ChondroFillerliquid*). It is a cell-free collagen implant composed of type I collagen designed for the immigration of cartilage and stem cells. *ChondroFillerliquid* is supplied as a two-chamber syringe. After injection into the defect zone, a dimensionally stable matrix equivalent to the product *ChondroFillergel* is formed within a few minutes. Due to its easier handling, it was used to fill the cartilage defects.

Initial studies have already shown positive results for the clinical outcome with the gel *ChondroFiller* [25–28]. MR tomographic MOCART (Magnetic Resonance Observation of Cartilage Repair Tissue) scores were even slightly better for *ChondroFiller* than for microfracture or MACT [26, 29].

In our in vitro study, the tested biomaterial did not have the expected effect and was not able to minimise the damage to the opposing cartilage. Presumably, the primary stability of the biomaterial was not sufficient, so that it was destroyed after only a few moves. The load was calculated according to the weight of the pigs so that it roughly corresponded to normal weight-bearing/standing with a stress rate of 0.42 MPa, that is generally considered to be rather low. In an animal model described by Spahn et al., permanent deformation of the hyaline cartilage only occurred at values of > 5 MPa, and fracture of the cartilage occurred from about 26 MPa [30]. In clinical practice, however, the biomaterial is usually not directly exposed to full weightbearing postoperatively because the biomaterial needs time for integration and tissue remodeling. For future tests, it would therefore probably be more appropriate to recalculate the level of axial pressure according to a partial load.

As mentioned above, the current literature describes predominantly the effects of cartilage damage to the biomechanical and biological properties of the adjacent, not to the opposite cartilage [9, 10]. There are also studies that have tested the effect of cartilage lesions on the integrity of the opposite cartilage using osteochondral cylinders. However, in one study, cartilage deformation was recorded and evaluated by video microscopy [19] and in another study, only the stress rates occurring during testing were measured [31]. Whether and to what extent a cartilage lesion can also lead to damage to the opposing cartilage has so far only been investigated in one study, in which osteochondral defects were placed in 8 minipigs and the opposing cartilage was examined after 12 months using MR tomography, histology and immunohistochemistry [32]. Significant lesions were found on the opposing cartilage, so that the authors concluded that full-thickness cartilage defects can lead to damage to the opposing cartilage. However, the effect of biomaterials was not tested.

### Limitations

The experiments were performed using porcine cartilage, not human cartilage. However, some studies have shown that the cartilage thickness as well as the water content and cell arrangement are very similar between porcine and human cartilage, and various tests on the stiffness did not show much difference [33–35].

Furthermore, bone-cartilage cylinders were tested in a machine with largely continuous loading. This is in contrast to normal gait, with increased and decreased loading sections of the cartilage. These physiological circumstances better allow the cartilage to dehydrate and rehydrate. It is possible that a reduced fluid content in our experiments may have led to a decreased load-bearing capacity of the cartilage. However, no damage was observed in the cartilage versus cartilage control group, and the 1 and 6 h analysis time points were short. Therefore, it is unlikely that hydration/dehydration processes had an effect.

## Conclusion

Our results show that cartilage lesions with sharp edges and larger, full-thickness cartilage defects with exposed subchondral bone can seriously damage the opposing cartilage. Furthermore, our study suggests that the primary stability of a biomaterial such as *ChondroFiller* does not withstand immediate full weight bearing, which should therefore be avoided immediately after implantation.
